# Associative and Identity Words Promote the Speed of Visual Categorization: A Hierarchical Drift Diffusion Account

**DOI:** 10.3389/fpsyg.2020.00955

**Published:** 2020-07-23

**Authors:** Lara Todorova, David A. Neville

**Affiliations:** Donders Institute for Brain, Cognition and Behaviour, Radboud University, Nijmegen, Netherlands

**Keywords:** response priming, drift diffusion model, motor control, response bias, processing speed

## Abstract

Words can either boost or hinder the processing of visual information, which can lead to facilitation or interference of the behavioral response. We investigated the stage (response execution or target processing) of verbal interference/facilitation in the response priming paradigm with a gender categorization task. Participants in our study were asked to judge whether the presented stimulus was a female or male face that was briefly preceded by a gender word either congruent (prime: “man,” target: “man”), incongruent (prime: “woman,” target: “man”) or neutral (prime: “day,” target: “man”) with respect to the face stimulus. We investigated whether related word-picture pairs resulted in faster reaction times in comparison to the neutral word-picture pairs (facilitation) and whether unrelated word-picture pairs resulted in slower reaction times in comparison to neutral word-picture pairs (interference). We further examined whether these effects (if any) map onto response conflict or aspects of target processing. In addition, identity (“man,” “woman”) and associative (“tie,” “dress”) primes were introduced to investigate the cognitive mechanisms of semantic and Stroop-like effects in response priming (introduced respectively by associations and identity words). We analyzed responses and reaction times using the drift diffusion model to examine the effect of facilitation and/or interference as a function of the prime type. We found that regardless of prime type words introduce a facilitatory effect, which maps to the processes of visual attention and response execution.

## Introduction

Words facilitate visual decisions in a variety of tasks. For example, the brief presentation of a word before a semantically related picture has been shown to facilitate the processing of the picture in a number of tasks such as detection of motion direction (Meteyard et al., 2007), word-picture matching (Boutonnet and Lupyan, 2015), recognition of ambiguous “Mooney” images (Samaha et al., 2018), and familiarity judgments (Amado et al., 2018). On the other hand, a word presented right before the picture can also lead to semantic interference, i.e., when a semantically unrelated word interferes with the judgment of an immediately following target compared to when the word is semantically related. In terms of behavioral performance, this effect translates to longer reaction times and/or more errors (Wentura and Degner, 2010) as has been shown to be the case in a variety of language tasks such as Stroop task(s), word–picture matching, and spoken-to-written word matching (Jefferies et al., 2008; Campanella et al., 2013; Faria et al., 2015). While facilitation effects have been interpreted as cognitive [with the locus of their influence being rooted either in the lexico-semantic system (Francken et al., 2015) or in the visual system (Boutonnet and Lupyan, 2015)] some of the interference effects have been associated with response processing. For example, in the Stroop task, the increased response latencies for naming the ink color of a conflicting color word (word: red, ink: green) in comparison to the non-conflicting (congruent) one (word: red, ink: red) are interpreted as a result of response conflict as opposed to informational conflict (Duncan-Johnson and Kopell, 1981; Cohen et al., 1990). Whether semantic facilitation and interference effects operate at the level of target and/or response processing is not yet entirely clear.

A paradigm, which is structurally similar to the Stroop task but less studied in terms of cognitive processes, is the response priming paradigm (Wentura and Degner, 2010). While Stroop-like effects appear at the response processing stage, response priming paradigms may tap into both target and response processing. In semantic priming paradigms, two stimuli are presented successively and participants are instructed to perform a task on the second stimulus (target), while the first stimulus (prime) is deemed to be non-relevant to the task (for example, participants might have to classify letter strings as words or non-words, with prime-target pairs being either semantically related or unrelated). In contrast, in response priming paradigms, primes are either congruent or incongruent with the response that has to be given to the target (*prime*: skirt, *target*: woman, *response*: woman, *task*: categorization). In the Stroop task, interference occurs when the ink color maps onto one response and the semantic meaning of the incongruent word to the other. In other words, interference arises due to the conflict between the responses. In response priming, similarly, interference occurs when the semantic meaning of the prime maps to one response and the semantic meaning of the target maps to the other response, leading to response conflict (De Houwer et al., 2002; Musch and Klauer, 2003). The fact that response priming and Stroop tasks are structurally similar does not exclude the possibility that interference effects could be explained by either one or both processes of response competition and target processing. It is therefore of theoretical importance to investigate semantic facilitation and interference effects in a response priming paradigm.

In this study, we focused precisely on this aspect and investigated in a response priming paradigm the effects of semantic facilitation and interference as reflected by changes in behavioral performance. We further explored whether facilitation and/or interference can be accounted for by mechanisms of response execution and/or target processing, which we formalized using the drift-diffusion model (DDM) (see further details in the next section). Participants in our study were asked to judge whether the presented (target) stimulus was a female or male face when the target was briefly preceded by a gender word (prime) either congruent (prime: “man,” target: “man”), incongruent (prime: “woman,” target: “man”) or neutral (prime: “day,” target: “man”) with respect to the face stimulus. Participants were instructed to decide about the gender of the target while ignoring the prime. We then looked at whether related word-picture pairs resulted in shorter reaction times in comparison to neutral word-picture pairs (facilitation) and whether unrelated word-picture pairs resulted in longer reaction times in comparison to neutral word-picture pairs (interference). We further examined whether these effects (if any) map onto response conflict or target processing. In order to shed light on the cognitive mechanisms underlying semantic facilitation and interference effects, we used a well-established approach from the cognitive modeling literature, the DDM (Usher and McClelland, 2001; Smith and Ratcliff, 2004; Ratcliff and McKoon, 2008).

### Drift-Diffusion Model

**FIGURE 1:**
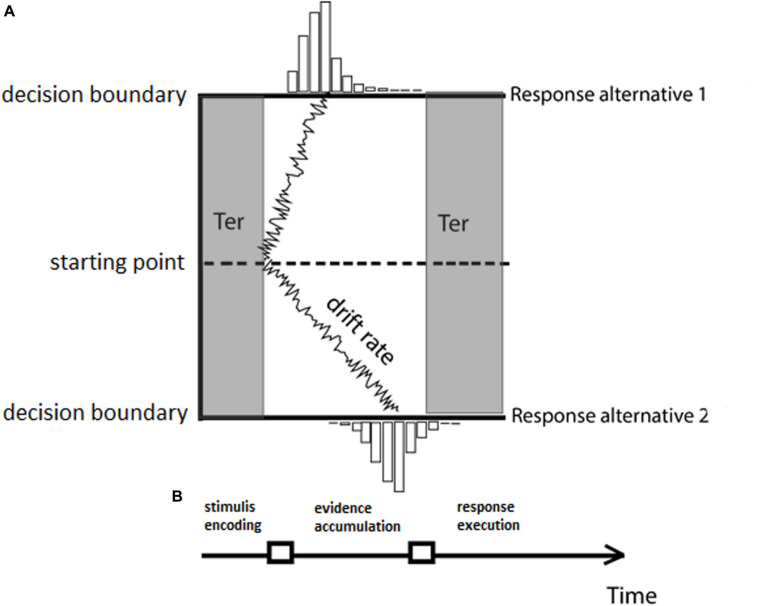
**(A)** The drift-diffusion model (DDM) with the four parameters: drift rate (*v*), boundary separation (*A*), starting point (*z*), and non-decision component (*T*_*er*_). **(B)** Stages of processing characterized by the DDM model. Different stages of processing are highlighted by the shadings in panel **(A)** indicating the mapping of the DDM components to the processing stages.

In the DDM approach, the process of making a decision about the gender of a face is described as the stochastic accumulation of sensory evidence over time toward one of two decision boundaries (male or female response, for instance). Once enough evidence is accumulated and one of the two decision boundaries is reached, the associated response is produced (for example, female). In the DDM, a total of four parameters describe the processing components underlying the decision-making process (see Figure 1): the rate at which evidence accumulates over time (*drift rate, v*), the amount of evidence that is necessary to produce a response (*boundary separation, A*), an optional *a priori* bias for a specific response (*bias, z*), and finally the time required to complete non-decision processes, such as motor preparation and/or stimulus encoding (*non-decision time, T_*er*_*).

The DDM model has been successfully applied to choice reaction time data in various experimental tasks (see, for reviews, Ratcliff and McKoon, 2008; Mulder et al., 2014), and the parameters recovered by the model have been shown to be well characterized in terms of cognitive processes (Voss et al., 2004). The drift rate or the speed of evidence accumulation is “determined by the quality of information extracted from the stimulus” (Ratcliff and McKoon, 2008). For example, in a color discrimination task, trials with less visible colors resulted in a lower drift rate as opposed to trials with the more visible colors (Voss et al., 2004). Crucially, the drift rate can be modulated by factors that are not only related to the stimulus being processed but are also related to contextual information associated with the study episode. For example, in a memory recognition task, a word that was studied three times had a higher drift rate than a word that was studied only once (Ratcliff and McKoon, 2008). Similarly, in an associative priming task, word primes that were associatively related to the target words resulted in a higher drift rate in comparison to word primes non-associatively related. It has been suggested that the drift rate in these contexts could represent “the quality of the match between a test word and memory” (Ratcliff and McKoon, 2008). The accumulation of the drift rate stops when either one of the two decision boundaries has been reached. The amount of evidence that is needed to make a decision is characterized by the boundary separation, that is, the distance of the boundaries from the starting point of the accumulation process, and it has been shown to be modulated by changes in task strategy (e.g., response caution). For example, when participants were instructed to prioritize response accuracy over response speed, changes in behavioral performance due to the adoption of a new response criterion by the decision maker were explained in the DDM model in terms of a higher value for the boundary separation parameter. The higher boundary separation translates to a longer period of information accumulation and, as a result of this, fewer errors being made by the decision maker (Bogacz et al., 2010). The starting point of the accumulation process instead reflects potential biases participants might have, which result in certain responses being “*a priori* more likely” (Mulder et al., 2014). For example, participants might, *a priori*, favor a “word” response in a lexical decision task (Wagenmakers et al., 2007). In addition, it was also shown that in a color discrimination task, a higher reward for a certain response resulted in participants adopting a starting point (*z*) closer to the decision boundary for the response with the higher value reward (Voss et al., 2004). Finally, the model component that does not account for decision processes is referred to as non-decision time. It reflects either stimulus encoding (which may not necessarily be perceptual encoding but rather access to memory in a memory task or lexical access in a lexical decision task, Ratcliff and McKoon, 2008) or the time required to execute a motor response. These contributions are combined together in one parameter (*T*_*er*_), which does not allow by itself the separation of encoding from response execution but rather allows for the separation of decision vs. non-decision processes. Studies that interpret *T*_*er*_ as an encoding or execution parameter use auxiliary methods such as neuroimaging to facilitate interpretations. For example, in an fMRI study investigating age-related performance in a visual search task, changes in non-decision time were associated with targets accompanied by response-incompatible distractors in the elderly group. *T*_*er*_ was correlated with the Frontal eye fields (FEF) and dorsal fronto-parietal regions, which suggested a major contribution from the visual encoding process (Madden et al., 2019). An Electroencephalography (EEG) study of figure-ground segregation instead found a correlation between N200 latency and the non-decision component, suggesting that N200 tracks the completion of visual encoding (Nunez et al., 2019).

In the DDM model, the parameters are combined non-linearly to enable inferences on the complete distribution of reaction time data. Technically, this is done by computing for each accuracy interval or bin (e.g., five intervals of 20% accuracy performance increments) the relative RT distribution and then fitting a (Gaussian) random walk model to each of the quantiles of the RT distribution. An intuitive way to think about how the parameters of the DDM model combine to produce RT distributions is the following. As an example, assume a hypothetical classification task where subjects have to classify a face as female or male and we assign the female face response to the upper boundary (Response alternative 1 in Figure 1) and the male response to the lower boundary (Response alternative 2 in Figure 1). Assuming there is no bias for either of the two response and the same boundary separation for both male and female face targets (same amount of evidence to be accumulated) and no difference in non-decision time, a drift rate toward the female-response boundary (i.e., positive drift) would indicate faster correct responses for face-related judgments of female faces. By contrast, differences in threshold or non-decision time would suggest that overall RTs are either longer or shorter in female-related judgments compared to male-related judgments (depending on the directionality of the difference), regardless of the correctness of the response. Similarly, a higher boundary separation would predict on average slower responses (since more information has to be accumulated), and thus a longer mean RT for a particular condition would be predicted. Whether a response would be correct or not, however, would be mainly driven by the drift rate. “Mainly” is used here because boundary separation (*A*), drift rate (*v*), and bias (*z*) together contribute to the generation of choice RT data, specifically to the differentiation of fast and slow responses in both correct and incorrect responses. For comprehension purposes, we point the reader to a simple qualitative taxonomy of the type of responses predicted by the DDM given a particular combination of *A* (boundary separation) and *v* (drift rate) parameters. If *A* is high and *v* is toward the correct response boundary (i.e., positive for upper boundary and negative for lower boundary), then the responses are predicted to be on average slow and correct. If, instead, *A* is high but *v* is toward the incorrect response boundary, then the responses are predicted to be on average slow and incorrect. f there is a change in the boundary separation parameter and, for example, if *A* is low and *v* is toward the correct response boundary, then the model predicts on average fast correct responses. If instead *A* is low and *v* is toward the incorrect response boundary, then the model predicts on average fast errors.

To sum up, in this study, we capitalize on the DDM model since it provides an ideal analytical tool for disentangling in the response priming task the cognitive processes involved in semantic facilitation and interference. Importantly, previous drift-diffusion studies revealed intriguing cognitive mechanisms underlying behavioral performance in language-related tasks that use the response priming paradigm. In the primed Stroop task, words-distractors were presented ahead of colored symbols that had to be categorized, and the facilitation effect was explained in terms of a change in boundary separation among congruent and incongruent conditions (Kinoshita et al., 2017). This result, however, could be explained in terms of stimulus probability, since a word prime was predictive of its matching response color (Kinoshita et al., 2017, p. 833). It has also been shown that predictive cue information results in boundary separation modulation (not drift rate) in a task where participants have to identify a house or face masked by noise, preceded by either a house or face cue with different degrees of reliability (Dunovan et al., 2014). The study of Kinoshita et al. (2017) is one of the recent studies on response priming that investigates language influences on target and response processing from the computational modeling perspective. Results are, however, not conclusive due to the predictability confounds described above. In this study, language primes were not predictive of the upcoming target, which allowed us to investigate the influence of language on facilitation and interference without introducing probabilistic confounds. Another interesting property that seem to affect participants’ performance in the response priming task, and which is crucial for this study, is the type of semantic relationship between prime and target, which will be discussed further in the following section.

### Type of Semantic Relationship

The type of semantic relationship between the target and the prime is another variable to be considered when investigating priming effects. For example, in the response priming paradigm, it was shown that associative and categorical primes involve different cognitive processes (Voss et al., 2013). While primes that belonged to the same category as the targets (i.e., categorical primes such as prime: lion, target: tiger) mapped onto the response execution stage, primes that were semantically associated with the targets (i.e., associative primes such as prime: king, target: crown) mapped onto the target processing stage. Specifically, the drift-diffusion analysis revealed that associative priming effects mapped onto the drift rate parameter, which indicated increased informational uptake for associative (prime: king, target: crown) word pairs in comparison to categorical ones (prime: lion, target: tiger). Furthermore, categorical primes mapped onto the non-decisional component, with congruent word-target pairs leading to a facilitation of the non-decisional processes, and incongruent ones resulting in interference. The authors explained categorical priming effects in terms of response competition and associative priming effects in terms of spreading activation (Collins and Loftus, 1975). Crucially, categorical congruency effects were associated with response competition processes regardless of the relevance of the congruency dimensions (i.e., whether the task was lexical decision or semantic categorization did not affect the results). On the contrary, Gomez et al. (2013) showed that categorical prime-target pairs mediated both the non-decision and the drift rate components. In the study of Gomez, however, the authors used variation of categorical primes involving identity primes (prime: house, target: house) instead of different words being related to each other categorically (such as prime: lion, target: tiger). Furthermore, they used a lexical decision task instead of the semantic categorization task used by Voss et al. (2013), which altogether might have led to differences in the experimental results. Other evidence from the word production literature shows a dissociation between associative and identity primes. For example, in picture–word interference, where participants name a picture and ignore a distractor word, picture naming is slower when the target image and distractor word are related in comparison to when they are unrelated (Glaser and Düngelhoff, 1984; Piai et al., 2013; Piai and Knight, 2018). Interestingly, other types of semantic relations such as for example associations, hypernym-hyponym, part-whole, or nouns-verbs (Lupker, 1979; Mahon et al., 2007; Kuipers and La Heij, 2008) result in facilitation or no modulation. Brain imaging studies have also shown a dissociation between associative and categorical relationships in terms of their neural bases (for a review, see Mirman et al., 2017). In sum, existing experimental evidence suggests that associations and categorical relations might have a differential effect on facilitation and interference and therefore should be properly accounted for in tasks that involve response and attention control. In this study, we specifically address the question of whether prime-target pairs that are related to each other either categorically (e.g., prime: “man,” target: man) or associatively (e.g., prime: “tie,” target: man) result in facilitation or interference effects and, if so, whether such effects occur at the level of response processing, target processing, or both.

### The Present Study

Our primary interest was verbal interference/facilitation in the response priming paradigm, either at the level of response execution or of target processing, when manipulating the type of semantic relationship linking prime and target. The facial features of the target pictures were morphed from male to female parametrically, and the task included ambiguous faces solely for participants’ engagement purposes. The primes were either associations (“tie,” “dress”) or identity primes (“man,” “woman”). In addition, primes were either congruent (prime: “man,” target: man), incongruent (prime: “woman,” target: man) or neutral (prime: “day,” target: man) with respect to the face. Participants were asked to decide about the gender of the target while ignoring the prime. Participants’ behavioral performance (responses and reaction times) was analyzed with the DDM approach to examine the effect of facilitation and/or interference as a function of the prime type.

The DDM approach focused specifically on testing two hypotheses. First, we investigated whether congruency effects in response priming tap into the cognitive mechanisms associated with the processing of the response and/or target. Under the response competition account, congruency effects (e.g., the prime maps onto a “female” response and the face is a female face) would map onto the speed of the non-decisional processes (i.e., slower for incongruent and faster for congruent). Under the target processing account, congruency effects would map onto the speed of processing of the target picture (drift rate). In this case, we expected the drift rate (*v*) to be higher in congruent vs. incongruent word-picture pairs. Second, we investigated whether the type of semantic relationship – associative or identity – would have an influence on the direction of the effect, i.e., facilitation or interference. For this purpose, we tested whether identity words (“man,” “woman”) and associative words (“beard,” “dress”) tap into response conflict processes (*T*_*er*_) and/or perceptual uptake (*v*). We furthermore tested whether identity words would enhance the rate of evidence accumulation as opposed to associative words and whether identity words would lead to faster motor preparation/execution in comparison to the associative ones.

To sum up, with this work, we aimed to investigate whether associative and identity words relating to gender categories affect cognitive processing, specifically the stages of response execution and/or information evaluation of the target during gender categorization.

## Materials and Methods

### Subjects

The study was approved by the local ethics committee (CMO Arnhem-Nijmegen, Radboud University Medical Center) under the general ethics approval. All participants provided written informed consent approved by Radboud University, Nijmegen. A total of 47 volunteers (23 females) recruited via the Radboud Research Participation System (native Dutch speakers, right-handed, age range: 19–35 years, mean age = 24.74, SD = 3.53) took part in the study. We performed two additional studies to develop and pre-test the materials. All participants reported no neurological disease and had normal or corrected-to-normal vision. All of the participants received monetary compensation for their participation.

### Target Pictures

**FIGURE 2:**
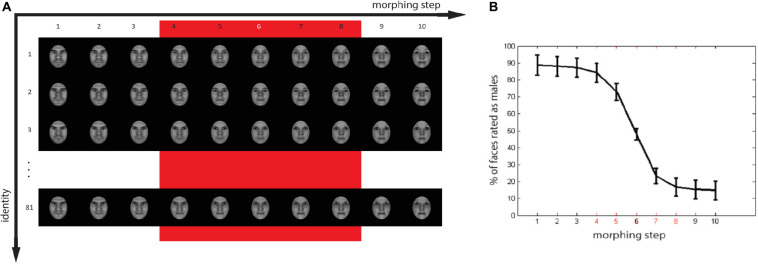
**(A)** Stimuli set with 81 artificially generated identities, each of which was morphed in ten steps across the male to female spectrum. The red square indicates the stimuli selected for the experiment. **(B)** Proportion of faces evaluated as males as a function of morphing step. Faces of morphing steps 4 (very male), 5 (less male), 6 (ambiguous), 7 (less female), and 8 (very female) were selected for the main experiment. Faces of step 6 were not included in the analysis [white marker in panel **(A)** and black in panel **(B)**].

A set of realistic 3D gender-morphed faces was created with the use of FaceGen Modeller 3.5 (Singular Inversions). The technical details of the computation method used by the software are discussed in Blanz and Vetter (1999). We created 81 Western face identities, for which we gradually morphed gender features from extremely female to extremely male in 10 equal steps (Figure 2A). Face stimuli were cropped to remove hair and ears and presented frontally. We controlled for luminance using the SHINE toolbox for MATLAB (Willenbockel et al., 2010).

### Target Picture Evaluation and Selection

In order to obtain subjective perceptual ratings of the face gender, we conducted a stimulus evaluation experiment where subjects performed a forced two-choice task on the gender of each of the faces of the experimental set. A separate pool of 47 volunteers (24 males, native Dutch speakers, age range 20–32 years, mean age = 23.83, SD = 2.88) participated in a separate experiment to evaluate the face stimuli. Another pool of volunteers (24 females, native Dutch speakers, right-handed, age range 20–53 years, mean age = 26.75, SD = 6.65) performed the semantic ratings of priming words. Each trial in the evaluation experiment started with a fixation cross that stayed on the screen for 1500 ms, after which the target was visually presented for 500 ms. Participants were able to deliver a response for an additional 2000 ms after the picture was removed from the screen. Based on the responses, we identified the morphing step of the faces that was perceived ambiguously. We included five morphing steps in the main experiment: the most ambiguous face in the middle of the continuum, plus two steps in either direction away from the middle point (Figure 2B). We define faces of morphing step 6 as ambiguous faces, and all the others as less ambiguous, with morphing step 4 as the most male face and morphing step 8 as most female face. The percentage of faces evaluated as males for ambiguous faces (step 6 in the Figure 1B) was 47.87% (SD = 16.45), for the selected extremely male faces (step 4), 84.21% (SD = 27.19), and for selected extreme-female faces (step 8), 16.80% (SD = 25.57).

### Prime Word Evaluation and Selection

The experiment consisted of two conditions. In the first (*identity*) condition, the labels “man” (English: *man*) and “vrouw” (English: *woman*) were used as primes. In the second (*associative*) condition, a set of gender-associated words such as “mascara” and “tie” were used as primes (see Supplementary Table S1.2). The words for this condition were preselected using the database from the “small world of words project” (De Deyne et al., 2013) and subsequently rated by naïve participants. In the rating experiment, the participants had to indicate for each word how related that word was to the words “male” or “female” on a 7-point scale (-3 = related to male; +3 = related to female). For half of the participants, female and male axes were swapped. Based on the rating outcomes, a selection of 40 words was made, which included 20 male-related and 20 female-related words. In addition, 20 words from the semantic category “furniture” (the neutral condition) and 20 catch words from diverse semantic categories were selected. The furniture and filler words were associated with neither the male nor the female categories according to the ratings. Male- and female-associated words were matched for word length, frequency per million, and concreteness (all *p* > 0.30). The catch trials were excluded from subsequent analyses. Frequencies for all words were extracted using the Subtlex corpus (Keuleers et al., 2010). The mean frequency, concreteness, and length of the materials used are indicated in Table 1.

**TABLE 1:** Characteristics of the words used in the priming experiment.

Associated word groups	Length	Frequency	Concreteness
female (*N* = 20)	*M* = 6(*SD* = 2.1)	*M* = 60.4(*SD* = 101.5)	*M* = 4.1(*SD* = 0.6)
male (*N* = 20)	*M* = 5.8(*SD* = 1.9)	*M* = 108.2(*SD* = 204.6)	*M* = 4.3(*SD* = 0.6)
neutral (*N* = 20)	*M* = 5.8(*SD* = 1.9)	*M* = 69.6(*SD* = 187.5)	*M* = 4.1(*SD* = 0.9)

### Semantic Similarity

It could be argued that the proposed associative words in the stimulus list we used contain both associations (“tie”) and identity-like words (“brother,” “father”). Therefore, we sorted the initial associative set into associations and labels (see Supplementary Table S1.2). Further, to control for the semantic similarity between the prime words (identity: “man,” labels: “father,” associative: “tie,” “dress”) and target concept (“male” introduced by a male face or “female” introduced by a female face), we used the *snaut* tool (Mandera et al., 2017) which is based on word2vec representations (Mikolov et al., 2013). The word2vec model represents words’ semantics as a vector of features, and the semantics of a certain word can be characterized by comparing the vector representations. The measure of semantic similarity we report here is cosine similarity, which has particular advantages over other measures such as Euclidean and Manhattan in cases where the vector magnitude matters. First, we calculated the semantic distance between each of the primes to the target concepts (associations: “beard” – “man,” labels: “father” – “man,” identity words: “man” – “man”) in terms of cosine distance (see Supplementary Table S1.2). Next, we tested whether the proposed word sets (associations, labels, identity words) differed in semantic measures using Bayesian ANOVA, which accounts for the non-equal number of words per word group. The null hypothesis states that there is no difference between the conditions of interest, whereas the alternative posits that the conditions of interest are different. A Bayes factor (BF) is defined as the ratio between the evidence in favor of the alternative hypothesis (H_1_) over the evidence in favor of the null hypothesis (H_0_), denoted by the subscript 10 in the Bayes factor abbreviation BF_10_. BFs estimate graded evidence in favor of or against the alternative hypothesis (Wagenmakers et al., 2018) and can be interpreted as follows: BF_10_ = 1–3 indicates “anecdotal” evidence for H_1_ compared to H_0_; BF_10_ = 3–10 indicates “moderate” evidence for H_1_ compared to H_0;_ BF_10_ = 10–30 indicates “strong” evidence for H_1_ compared to H_0;_ BF_10_ = 30–100 indicates “very strong” evidence for H_1_ compared to H_0;_ BF_10_ > 100 indicates “extreme” evidence for H_1_ compared to H_0_. Bayesian ANOVA was carried out using JASP (JASP Team, 2018).

**FIGURE 3:**
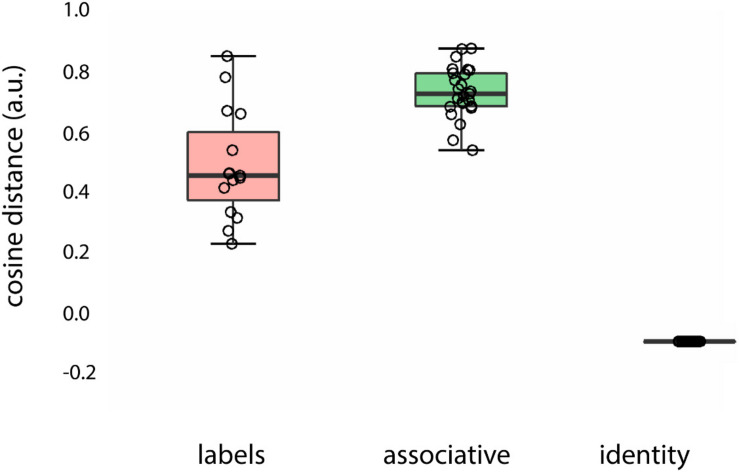
Semantic distance between prime words (labels, associations, and identity words) and target concept introduced by the face (“female”/“male”) color-coded for type of prime word: red, labels (“father”/“daughter”); green, associations (“beard”/“skirt”); black, identity words (“man”/“woman”).

The results of the analysis are presented in Figure 3. Words of different types, fully overlapping with the picture (“man,” “vrouw,” i.e., identity), less overlapping (“father,” “sister,” i.e., labels) and associative items (“tie,” “dress”), translated to different semantic distances from the target concept (main effect of semantic distance, BF10 = 1.297e + 47). Specifically, identity words (“man/vrouw”) had higher similarity (lower cosine distance) to the target concept (“man,” “vrouw”) in comparison to the partially overlapping words (e.g., “father,” “sister”), BF10 = 3.707e + 6. Associative words were further from the target concept in comparison to the identity words, BF10 = 2.548e + 22, whereas labels were closer to the target concept in comparison to the associative words, BF10 = 250.437.

The semantic distance between identity words and labels was larger (evidenced by a “very strong” BF) than between labels and associative words (labels – associations > labels – identity words: BF_10_ = 2.548e + 22).

Therefore, for the first set of analyses (see section “Congruency Analysis”), we collapsed labels and associations (the non-identity condition), especially in light of the fact that in the current paper we did not focus on the various types of similarity between the words but rather focused on the contrast between identity words and all other words that point to the same concept but have larger semantic distances.

However, since the difference between the labels and associations is statistically significant, we provide additional analyses excluding the labeling primes and thus only comparing associative and identity primes. These additional analyses are explained further below (see the “Behavioral Analysis” section, and section “Secondary Congruency Analysis”: associative versus identity primes).

### Procedure

**FIGURE 4:**
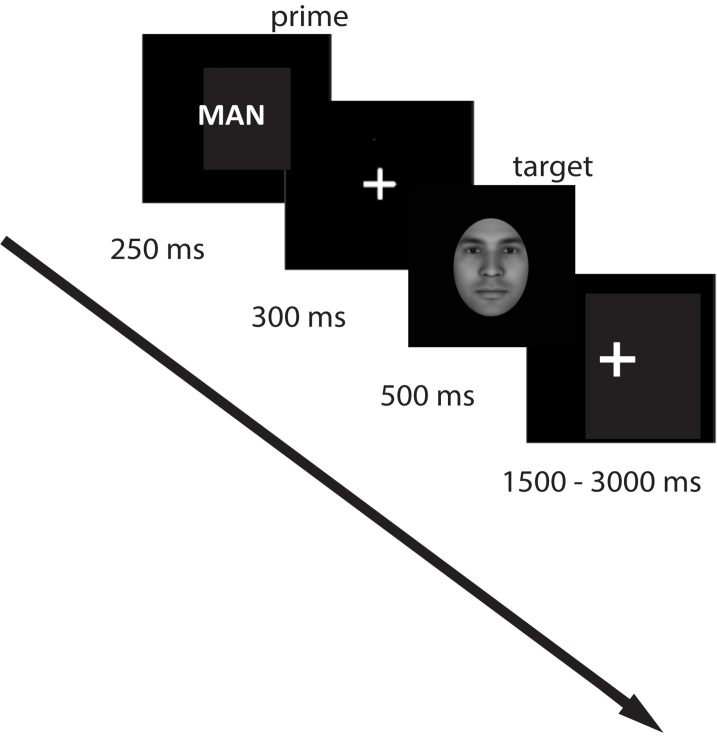
Experimental design. A prime (label or word associated with the target concept) was presented for 250 ms, followed by a fixation cross (300 ms), after which the target picture was presented, followed by a jittered fixation cross for 1500–3000 ms. Participants had to indicate their decision on the gender of the presented face by button press.

Participants were seated in a comfortable chair in front of a computer screen in a sound-protected room. On each trial, a prime word was presented for 250 ms, after which a fixation cross remained on the screen for 300 ms. The visual target was presented for 500 ms, followed by a jittered inter-trial interval of 1500–3000 ms (Figure 4). The total number of trials was 800. The experiment was divided into two blocks (400 trials per each block) with an optional break between the blocks. Overall, for the associative prime condition, there were 20 male-related, 20 female-related, and 20 neutral words. For identity words, we had the word “woman”/“man” presented 20 times each, and 20 neutral words (the same as we used in the associative condition). The words were presented with each morphing step (face identity was shuffled with no repetition), and each prime word was repeated five times. The trial order was randomized. The total duration of the experiment was approximately 90 min.

### Task

Participants were instructed to decide upon the gender (male or female) of the face based on the image presented and to respond with a keyboard button press (middle/index finger; the mapping of the response buttons was counterbalanced across participants). Participants had up to 2 s to respond after the onset of the picture and were instructed to skip the trials on which the prime belonged to the category “furniture.” This “go/no-go” task ensured that participants read the prime word.

### Analyses

#### Behavioral Analysis

We investigated priming effects by separately analyzing measures of behavioral performance: reaction times (RT) and choice responses. We performed a 2 by 3 repeated measures analysis of variance (rm ANOVA) with *prime type (identity* or *associations)* and *congruency (congruent, incongruent, neutral)* as factors. We preselected congruent (target: male face, prime “man”; target: female face, prime “female”), incongruent (target: male face, prime “female”; target: female face, prime “man”), and neutral (target: male face, prime “day”; target: female face, prime “day”) word-target pairs collapsing across very- and less-gendered morphing steps (step 4, 5; step 7, 8). An arcsin transformation was applied to the percentage of correct responses before entering it into the ANOVA. We performed *post hoc* comparisons for main effects using Holm correction. Morphing step 6 was excluded from all analyses since ambiguous faces can require a different configuration of the decision process in comparison to male- and female-gendered faces. Whereas faces of step 6 are marked by high-uncertainty, faces of steps 4, 5, 7, and 8 are instead marked by low uncertainty. Given this potential difference and given our focus on the potential differences among types of word primes, we decided to exclude step 6 items to avoid potential confounds in terms of uncertainty. It could be argued that the associative condition introduced in this study may be viewed as a conjunction of “labels” and “associations,” which would make the comparison between the two groups confounded (see section“Semantic Similarity”). Thus, in a secondary set of analyses, we repeated a 2 by 3 repeated-measures (rm) ANOVA analysis on the associative words, omitting potentially confounded words in the non-identity condition (i.e., the 15 “label primes” (e.g., father) and resulting in 25 associative words, see Figure 3 and Supplementary Table S1.2) using the Bayesian approach, which accounts for the unequal number of trials.

Moreover, it could be argued that facilitation/interference effects can be modulated by repetition of the materials (i.e., the identity primes are repeated more than associative primes); therefore, we also performed an analysis of repetitions. First, we calculated the number of repetitions for the conditions of interest (congruent, incongruent, and neutral) separately for associative and identity words. We then calculated the average effect of interference (unrelated–neutral) and facilitation (related–neutral) for associations and identity words. Since the number of repetitions was unequal per condition for identity words (∼ 80 repetitions) and for associative words (∼ 50 repetitions), we calculated the facilitation/interference effect across the whole session with a step augmentation of 10 trials in order to better illustrate the differences between the identity/associative conditions. Further, we performed four ANOVAs (separately for interference and facilitation per associations and identity words) with the number of trial repetitions as the dependent variable. Additionally, to investigate the effect of prime type and prime gender on face gender categorization, we performed rm ANOVA (both RTs and responses) with *target face* (male or female), *prime gender* (male-related, female-related, or neural), *prime type* (association or identity) as within-subject factors. Finally, to investigate the role of the subjects’ sex on gender categorization, we ran a rm ANOVA with *target face* (male or female), *prime gender* (male-related, female-related, or neural), *prime type* (association or identity) as within-subject factors and subject’s *sex* (female, male subjects) as a between-subjects factor. The analyses were performed using JASP (JASP Team, 2018).

### Hierarchical Drift-Diffusion Model

In order to gain insights into the processing components underlying categorization in the identity and associative conditions, we analyzed choice reaction time data with the hierarchical DDM. The analysis was implemented in the Python toolbox HDDM 0.6.0 (Wiecki et al., 2013). One of the main advantages of the hierarchical Bayesian framework is that the simultaneous estimation of the model parameters at both the single-subject and group levels enhances statistical power since fewer trials are required to recover the parameters and the estimates are less susceptible to outliers (Wiecki et al., 2013), making it an appropriate analytic approach for the present study. Models with different combinations of free parameters were fitted to the data via Markov Chain Monte Carlo (MCMC) fitting routines. We used an accuracy coding scheme for the responses for congruent/incongruent/neutral prime-target pairs with the upper boundary reflecting a correct face categorization and the lower boundary an incorrect one. We defined the model space by allowing the parameters to vary freely over the factors of interest (*congruency, prime type*) of the experimental design (see Supplementary Table S2).

For each model, we evaluated the rate of convergence of the numerical fitting routines and then the ability of the model to capture the observed RT distributions. Models that failed to reach convergence or failed to capture the observed RT distributions were excluded from further analyses. Finally, the remaining models were compared against each other by computing the relative Deviance Information Criterion (DIC), which is a measure of the goodness of the model fit to the data that penalizes for the complexity of the model (Schwarz, 1978). A rm ANOVA was then used to test for significant differences in the parameter estimates of the best-fitting model and to quantify the evidence in support of a given hypothesis.

Finally, to investigate whether the sex of subjects could modulate the cognitive processes related to gender categorization (e.g., female subjects could have had a general inclination toward categorizing a face as a female), we stratified the bias structure (*z* parameter) according to the subject sex in a separate set of models, this time using a stimulus coding scheme. We reasoned that females could be more accurate in face categorization in comparison to males, which would be reflected in different amounts of *a priori* information being available before each decision (the starting point could be closer to the female response boundary). To test for this hypothesis, we compared the following HDDM models: a model with no bias included (model 33), a model testing whether males and females have different *a priori* information guiding the categorical decisions (model 32), and the current winning model (model 30), which included bias with no gender differentiation. We report the results in section 2.0.

## Results

### Behavioral Analyses

#### Congruency Analysis

**FIGURE 5:**
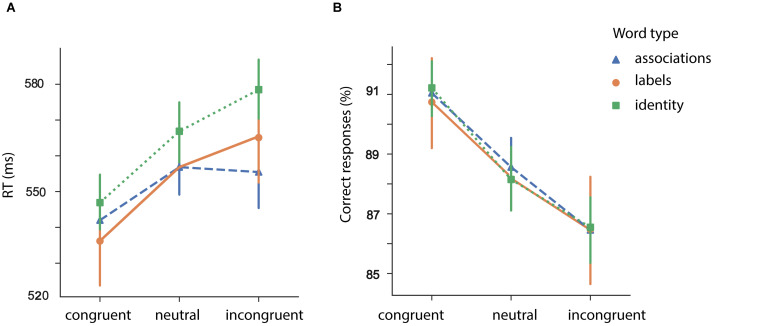
Mean reaction times **(A)** and correct responses **(B)** for identity (“man”/“woman”) and associations [mix or associations (“beard”/“skirt”) and labels (“father”/“daughter”) as a function of semantic relatedness]. Error bars represent 95% confidence interval (CI); RT, response time.

Behavioral results are summarized in Figure 5. We highlight that the associative condition consists of both labels (“father”/“daughter”) and “pure” associations (“beard”/”skirt”). For the rationale, see section “Semantic Similarity.” Participants’ speed of response varied as a function of congruency (main effect of *congruency*: *F* (2, 92) = 7.63, *p* < 0.001). On average, congruent words resulted in faster RTs in comparison to incongruent ones (congruent > incongruent: *t* = −6.13, *p* < 0.001, SE = 0.004, Mean difference = −0.027) and to neutral ones (congruent > neutral: *t* = −4.86, p < 0.001, SE = 0.004, Mean difference = −0.021). We also found that the incongruent words resulted in slower RTs in comparison to neutral ones (incongruent > neutral: *t* = 2.56, *p* = 0.04, SE = 0.002, Mean difference = 0.006).

We further found that participants’ performance varied depending on the type of the prime [main effect of *prime typ*e: *F* (1, 46) = 4.70, *p* = 0.035]. On average, identity words resulted in longer RTs in comparison to associative words (associative > identity: *t* = −2.16, *p* = 0.035, SE = 0.007, Mean difference = −0.014).

Congruent pairs with associative and identity words resulted in shorter RTs in comparison to neutral pairs (congruent > neutral (identity): *t* (46) = −3.53, *p* < 0.001; congruent > neutral (associations): *t* (46) = −5.07, *p* < 0.001). Interestingly, only incongruent pairs with labels resulted in longer RTs in comparison to the pairs with neutral words (incongruent > neutral (identity): *t* (46) = 2.32, *p* = 0.024; incongruent > neutral (associations): *t* (46) = 0.40, *p* = 0.689).

For the proportion of correct responses, we did not find a difference for *prime type* (identity/associations): *F* (1, 46) = 0.014, *p* = 0.90. The effect of congruency did reach significance: *F* (2, 92) = 23.48, *p* < 0.001. We did not find an interaction between *prime type* and *congruency*: *F* (2, 92) = 0.13, *p* = 0.87.

#### Secondary Congruency Analysis

We further excluded labels (“father”/“daughter”) from the associations (“beard”/“skirt”) and repeated the analysis in section “Congruency Analysis.” For the RT, we found a main effect of *congruency*: *F* (2, 92) = 22.53, *p* < 0.001. On average, congruent prime-pairs resulted in faster RTs in comparison to incongruent word-pairs (*t* = −5.42, *p* < 0.001, SE = 0.004, Mean difference = −0.023) and to neutral words (*t* = −4.63, *p* < 0.001, SE = 0.004, Mean difference = −0.019). There was no difference between unrelated and neutral word-target pairs: *t* = 1.78, *p* = 0.081, SE = 0.002, Mean difference = 0.004.

We also found that there was a main effect of *prime type*: *F* (1, 46) = 5.10, *p* = 0.029. On average, associations resulted in shorter RTs in comparison to identity words: *t* = −2.25, *p* = 0.029, SE = 0.006, Mean difference = −0.014.

We also found an interaction between *congruency* and *prime typ*e: *F* (2, 92) = 5.45, *p* = 0.006. To investigate the interaction, we performed a series of pair-wise *t*-tests with the following results: both related associative and related identity primes resulted in shorter RTs in comparison to the neutral primes [congruent > neutral for associations: *t* (46) = −3.959, *p* < 0.001; congruent > neutral for identity: *t* (46) = −3.537, *p* < 0.001]. As for the incongruent condition, only identity pairs resulted in interference [incongruent > neutral for identity: t (46) = 2.328, *p* = 0.024; incongruent > neutral for associative: t (46) = −0.82, *p* = 0.413].

For the proportion of correct responses we found a main effect of *congruency*: *F* (2, 92) = 19.58, *p* < 0.001. We did not find a main effect of *prime typ*e: *F* (1, 46) = 0.002, *p* = 0.96, or an interaction effect between *congruency* and *prime type*: *F* (2, 92) = 0.084, *p* = 0.92.

#### Analysis of Repetitions

**FIGURE 6:**
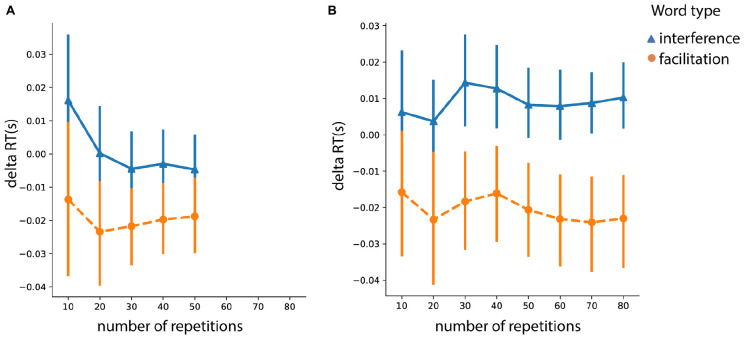
Interference and facilitation effects across trials for **(A)** associative words (“beard”/“skirt”) and **(B)** identity (“man”/“woman”) words. Error bars represent 95% confidence interval (CI); RT, response time.

Since there was intrinsically a different number of trials for associative vs. identity words, we performed a repetition analysis with the purpose of investigating the facilitation and interference effects as a function of the number of trials. The results are presented in the Figure 6 for associative (Figure 6A) and identity (Figure 6B) words. To reiterate, we defined the facilitation effect as the difference between congruent and neutral prime-target pairs. The interference effect was defined as the difference between incongruent and neutral pairs. There were, on average, 80 repetitions for identity primes (for each of the related and unrelated conditions) and 50 repetitions for associative primes (for each of the congruent and incongruent conditions) per subject.

Within each of the conditions – associative or identity – we tested the effect of repetition separately for interference and facilitation effects. For the identity condition, we did not find a repetition effect for either facilitation (*F* (7,322) = 0.96, *p* = 0.46) or interference (*F* (7,322) = 0.91, *p* = 0.49). For the associative condition, the repetition effect did not modulate the facilitation effect (*F* (4, 184) = 0.71, *p* = 0.58). However, the interference effect was affected by the repetition effect (*F* (4,184) = 4.39, *p* = 0.002). This was mainly driven by a larger interference effect for the first 10 trials. Specifically, analysis of repeated contrasts showed significant difference only between 10 vs. 20 trials (*t* = 2.69, *p* = 0.008, SE = 0.006, Estimate = 0.016) but neither for 20 vs. 30 (*t* = 0.79, *p* = 0.42, SE = 0.006, Estimate = 0.005) nor for other repeated contrasts.

To sum up, the analysis investigating the effects of *congruency* and *prime type*, even when accounting for the potential confounds in the prime type, showed consistent results. Particularly, both identity words and associations resulted in a facilitation effect but only identity words produced an interference effect. However, as we show in Figure 6A, the repetition of items did affect the associative words, which reduced after 20 trials.

#### Analysis of Prime Effects on Gender Categorization

**FIGURE 7:**
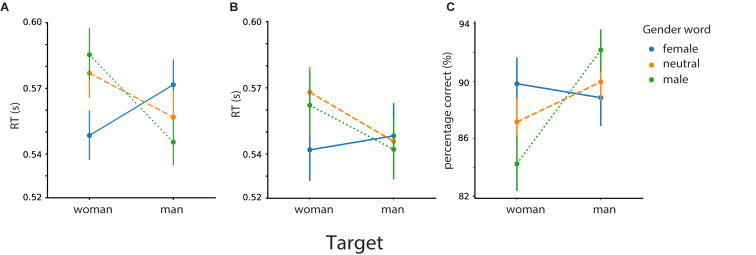
Mean reaction times for gender categorization of the target as a function of identity words (“man”/“woman”) **(A)** and associations (“beard”/“skirt”) **(B)**. Percentage of correct responses **(C)**. Error bars represent 95% confidence interval (CI); RT, response time.

To investigate the effect of prime type and prime gender on face gender categorization, we performed a 2 by 3 by 2 rm ANOVA (both RTs and responses) with *target face* (male or female), *prime gender* (male-related, female-related, or neural), *prime type* (association or identity) as within-subject factors. The results of this analysis are presented in Figures 7A–C.

For the RTs (Figures 7A,B), we found a main effect of *target gender* (male vs. female faces) [*F* (1,46) = 7.27, *p* = 0.01], a main effect of semantic relatedness of the prime with the target gender (male/female/neutral prime) [*F* (2,92) = 6.13, *p* = 0.003], and a main effect of *prime type* (identity or associative word) [*F* (1,46) = 5.05, *p* = 0.02]. Most importantly, we found a tree-way interaction between *target face*, *prime gender*, and *prime type* [*F* (2,92) = 6.32, *p* = 0.003].

For both identity words and associations, participants’ responses were faster when a female face was preceded by a female word in comparison to a male-related word [identity (male vs. female): *t* (46) = 5.21, *p* < 0.001; associations (male vs. female): *t* (46) = 2.84, *p* = 0.007] and to neutral words [identity (neutral vs. female): *t* (46) = 4.37, *p* < 0.001; associations (neutral vs. female): *t* (46) = 4.01, *p* < 0.001]. We did not find differences in performance for female faces preceded by male primes in comparison to neutral primes [identity (male > neutral): *t* (46) = 1.05, *p* = 0.149; associations (male > neutral): *t* (46) = −1.04, *p* = 0.84]. Altogether, this suggests facilitation rather than interference effects for female faces, regardless of identity or associative words.

As for the male target faces, in case of identity primes, participants were faster when it was preceded by the male prime in comparison to the female prime but not in comparison to the neutral primes [identity (female vs. male): *t* (46) = 3.62, *p* < 0.001; identity (neutral vs. male): *t* (46) = 1.68, *p* = 0.05]. For male faces, we do not find enough evidence in favor of facilitation or interference.

We present the results of the analysis focusing on the response in Figure 7C. As for responses, we found a significant interaction between target face and prime gender [*F* (2, 92) = 18.92, *p* < 0.001]. Particularly, participants were more accurate for female faces when preceded by female words in comparison to male words [female > male: *t* (46) = 4.45, *p* < 0.001] and neutral words [*t* (46) = 3.47, *p* = 0.001]. With regards to the male faces, participants were more accurate in classifying them when preceded by male words in comparison to female words [*t* (46) = 4.24, *p* < 0.001) and neutral words [*t* (46) = 2.99, *p* = 0.004].

### Hierarchical Drift-Diffusion Modeling

Next, we conducted a drift-diffusion analysis using RTs and choice responses from the associative and identity conditions (following the analysis described in section “Secondary Congruency Analysis”).

### Model Convergence and Model Fit

For all of the analyses reported, the MCMC (Gelman and Rubin, 1992) fitting routines were run for 25,000 iterations with a burn-in period of 10,000 iterations and a thinning of 1. Model convergence was assessed by examination of the posterior samples and of the R-hat statistic, which is a measure of convergence among multiple MCMC chains (three for the present study). Posterior density estimates, which are stable over multiple samples, indicate that the fitting routines have converged to a fixed estimate. An R-hat statistic below 1.1 indicates that chains with different starting values have converged to the same posterior estimate. Successful convergence was confirmed by an MCMC error for all of the parameters smaller than 0.01. We further performed a comparison between observed and recovered RT distributions produced by the model (see Supplementary Figure S1). After assessing convergence, we carried out a quantitative comparison of alternative models by computing the associated DIC score for each model. DIC is a measure of the goodness of fit of the model to the data that is penalized for the complexity of the model, and therefore a model with a lower DIC score is to be preferred over an alternative model with a higher DIC score as the most parsimonious explanation of the data. Models that did not reach convergence were discarded and not included in the DIC comparisons.

Below, we report modeling results using behavioral data from congruency analysis (see section “Secondary Congruency Analysis”) and analysis of prime effects on face categorization (from section “Analysis of Prime Effects on Gender Categorization”). For the congruency analysis, the model that best described the data (i.e., the model with the lowest DIC score) was the model with the following parameters estimated per subject: drift rate (*v*) free over *congruency* and *prime type*, threshold (*A*) free over *prime type*, and non-decision time (*T*_*er*_) free to vary across *congruency* and *prime type* (see Supplementary Table S2 for details). Conventionally, a DIC difference of more than 10 indicates that the evidence in favor of the winning model is substantial (Burnham et al., 2002). Because the difference between the winning model (model 6, DIC −16601.7) and the second-best model (model 12, DIC −16588.0) exceeds 10 (13.7), we consider this evidence sufficient to select model 6 as the most parsimonious account of the data, and, therefore, further analyses focus on this model.

For the analysis of prime effects on face categorization, the model that best described the data had the following parameters estimated per subject: drift rate (*v*) free over *prime gender* and *target face*, boundary separation (*A*) free over *prime type* and *target face*, and non-decision time (*T*_*er*_) free over *prime gender*, *prime type*, and *targe*t *face* (see Supplementary Table S3 for details). Since the difference between the winning model (model 30, DIC −17677.7) and the second-best model (model 24, DIC −17627.3) exceeds 10, we consider this evidence satisfactory for selecting model 30 as the most parsimonious account of the data, and, therefore, further analyses focus on this model.

Finally, to investigate whether the sex of subjects could modulate cognitive processes related to gender categorization, we stratified the bias structure according to the subject sex. We reasoned that females could be more accurate in face categorization in comparison to males, which would be reflected in information available *a priori*. We found that both models with bias included (model 30A, DIC −17674.72, and model 30, DIC −17677.69) explain the data better in comparison to the model without bias included (model 30B, DIC −17382.4268). However, we did not find enough evidence to postulate that there is a difference in male and female bias settings (the DIC of model 30 does not exceed the difference of 10 in comparison to model 30A). See a short summary in Supplementary Table S4.

### Model Parameter Analysis

#### Congruency Analysis for HDDM Parameters

**FIGURE 8:**
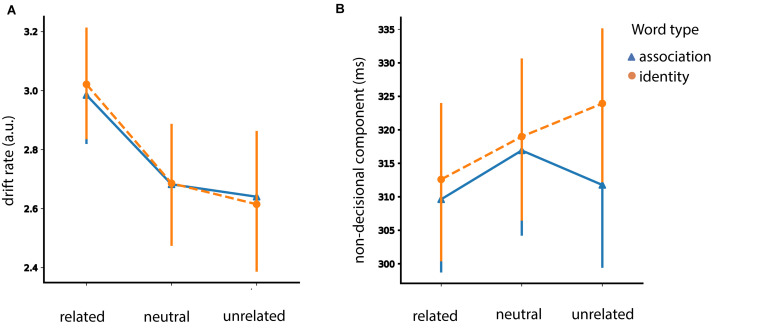
Congruency analysis. Posterior estimates of the hierarchical drift-diffusion model for the drift rate (*v*) and non-decisional parameter (*T*_*er*_). Error bars represent 95% confidence interval (CI).

The results of the modeling analysis are summarized graphically in Figure 8.

##### Drift rate

We found a *congruency* effect [*F* (2, 92) = 27.34, *p* < 0.001]. Particularly, congruent pairs had an increased drift rate in comparison to incongruent ones (*t* = 5.59, *p* < 0.001, SE = 0.067, Mean difference = 0.376) and in comparison to neutral ones (*t* = 6.88, *p* < 0.001, SE = 0.046, Mean difference = 0.0319). We did not find a difference between incongruent and neutral pairs (*t* = −1.168, *p* = 0.249, SE = 0.048, Mean difference = −0.056).

##### Boundary separation

We did not find a *congruency* effect [*F* (1, 46) = 2.63, *p* = 0.11].

#### Non-decision Component

We found an effect of *congruency* [*F* (2, 92) = 5.81, *p* = 0.004]. Congruent word-picture pairs had faster non-decisional time in comparison to incongruent (*t* = −2.78, *p* = 0.023, SE = 0.002, Mean difference = −0.007) and neutral (*t* = −2.92, *p* = 0.016, SE = 0.002, Mean difference = −0.007) pairs. There was no difference in the non-decision time between incongruent and neutral pairs (*t* = −0.049, *p* = 0.96, SE = 0.002, Mean difference = −1.050e-4). We found neither a main effect of *prime type* [*F* (1, 46) = 2.06, *p* = 0.15] nor an interaction between *congruency* and *prime type* [*F* (2, 92) = 2.74, *p* = 0.069].

To sum up, we found a congruency effect for the drift rate (*v*) and the non-decisional parameter (*T*_*er*_) but not for the decision boundary (*A*).

#### HDDM Representation of Priming Effects on Face Gender

**FIGURE 9:**
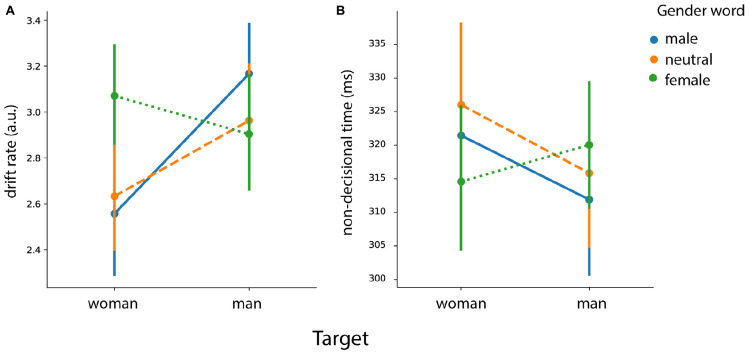
Gender priming effects analysis. Posterior estimates of the hierarchical drift-diffusion model for **(A)** the drift rate (*v*) and **(B)** the non-decisional parameter (*T*_*er*_). Error bars represent 95% confidence interval (CI).

The results of the modeling analysis are summarized graphically in Figure 9.

##### Drift rate

For drift rate, we found main effects of *target face* [*F* (1, 46) = 4.55, *p* = 0.038] and of *prime gender* [*F* (2, 92) = 12.70, *p* < 0.001]. We also found an interaction between *target face* and *prime gender* [*F* (2, 92) = 22.17, *p* < 0.001]. Results showed a higher drift rate for female faces preceded by female words in comparison to male words [*t* (46) = 5.96, *p* < 0.001] and in comparison to neutral words [*t* (46) = 6.03, *p* < 0.001). As for the male faces, the results showed an increased drift rate for male faces with male words in comparison to male faces with neutral words [*t* (46) = 4.36, *p* < 0.001) and female words [*t* (46) = 4.18, *p* < 0.001].

We did not find any effects when including sex as a between-subjects factor.

##### Boundary separation

We did not find any significant effects. Inclusion of sex as a between-subjects factor did not lead to any significant effects either.

##### Non-decision component

We found an interaction effect between *prime gender* and *target face* [*F* (2, 92) = 7.45, *p* < 0.001]. Results showed a faster non-decisional time for female faces preceded by female words in comparison to female faces preceded by male words [*t* (46) = 2.51, *p* = 0.015] and in comparison to the ones preceded by neutral words [*t* (46) = 3.04, *p* = 0.004]. As for the male faces, the results showed a faster non-decisional time when the target was preceded by the male prime in comparison to the female prime [*t* (46) = 2.70, *p* = 0.009]. However we found neither facilitation [neutral > male: *t* (46) = 1.32, *p* = 0.19] nor interference [male < neutral: *t* (46) = −1.33, *p* = 0.18] effects.

## Discussion

In this study, we investigated whether the different types of semantic relationships (Wentura and Degner, 2010; Mirman et al., 2017) (associations or identity words) result in facilitation and/or interference in response priming of a gender categorization task. Participants had to decide about the gender of presented faces after having seen a word prime. From the analysis of reaction times, we found that both identity (e.g., prime “man”) and associative (e.g., prime “beard”/“father”) words resulted in a facilitation effect (congruent vs. neutral), whereas only identity words resulted in interference (incongruent > neutral). We further combined RTs and choice responses within the analytical framework of the DDM with the purpose of investigating the cognitive processes underlying facilitation and/or interference. We found a facilitation effect in both associative and identity words that translated to modulations in drift rate and non-decisional time.

### Congruency With the Target Words Facilitates Information Processing of the Target Picture

Words are one of the top-down factors (such as reward or task strategy) that influence perceptual decisions. Indeed, it has been shown that a larger reward for one of the response options or increased likelihood of occurrence of two events results in an enhanced starting point of evidence accumulation for that particular response (Mulder et al., 2012). It has recently been proposed that language affects perception by setting predictive priors that sharpen perceptual representations (Simanova et al., 2016). Kinoshita et al. (2017) indeed showed that the brief presentation of a color word followed by the presentation of a color sign to be categorized resulted in a facilitation effect that translated to boundary separation and starting-point modulations. This prompted the idea that words do indeed affect perceptual decisions in a predictive fashion. However, this could be attributable to the internal statistics of the experiment – words were predictive of the upcoming color of the target. In our experiment, we unambiguously show that when words are non-predictive of upcoming features of the target stimuli, modulations in neither threshold nor starting point are manifested. Instead, we found that words result in an increased drift rate, which we interpret in terms of faster target processing speed.

Our finding supports well-established facilitation effects of language on perceptual decisions. For example, it has been shown that language can speed up recognition of visually presented objects (perceptual sensitivity or d’ prime), as has been demonstrated for the identification of facial expression (Carroll and Young, 2005) and for the detection of motion direction (Meteyard et al., 2007). Effects of language on visual perception have been demonstrated across different tasks and perceptual domains, including color categorization (Gilbert et al., 2006, 2008; Winawer et al., 2007) and face recognition (Landau et al., 2010; Anderson et al., 2014). Experimental studies in the language domain are prone to interpret perceptual sensitivity results in terms of perceptual advantage, i.e., language tapping into the low-level representations (Meteyard et al., 2007). However, the theoretical premises of the drift rate describe it as a post-encoding measure that does not reflect the low-level encoding (of a target picture) but rather reflects an intermediate processing stage between stimulus encoding and response execution.

Usually, in priming studies, both prime and target are words, and the modulations in drift rate are therefore interpreted in terms of increased spreading activation of a lexico-semantic nature (Voss et al., 2013). However, in cross-domain priming (prime: word, target: picture), the properties of the visual stimulus might change the nature of the process reflected by the drift rate. For example, it has been shown that the physical strength of the stimulus (i.e., intensity) was captured by a late event-related EEG potential, the centro-parietal positivity (CPP), that also tracked subjective perceptual experience (above the physically presented evidence) (Tagliabue et al., 2019). Another study showed that the rate of evidence accumulation is correlated with the P300 component, which scaled with target detection difficulty (Twomey et al., 2015) and indexed the duration of stimulus evaluation processes (Kutas et al., 1977; Duncan-Johnson, 1981).

Together, these studies suggest that the modulation of evidence accumulation can reflect a separate meta-process. This could be tested by looking at whether the advantage in processing speed is due to lexico-semantic, visual, or meta-processing facilitation, for example, using EEG. One would expect a modulation of either the N400 (see, for a review, Kutas and Federmeier, 2011) in case of a semantic advantage, a P300/CPP modulation in case of an attention advantage, or a P1 modulation in case of early visual advantage (Boutonnet and Lupyan, 2015). Regardless of the exact nature of the drift modulation, we show that the priming effects modulate the informational processing of the picture. To clarify which type of information is needed and, as a consequence, is reflected in the drift rate requires a combination of mathematical modeling and neuroimaging tools, which may be of use for future studies.

### On the Processing of Associations and Identity Words

We found that associative and identity words differ in the magnitude of RTs: longer for identity words in comparison to associative ones (no difference was found in accuracy data). It is known that upon repeated presentations of an item, the chance of an error can be both diminished (repetition priming) and increased, resulting in cumulative semantic interference (Oppenheim et al., 2007). For example, in a continuous naming paradigm, subjects name pictures that belong to different categories, and the naming times increase linearly with the number of pictures belonging to that category. Interestingly, the repetition of an item produced the same cumulative interference effect as additional novel exemplars in the category (Navarrete et al., 2010). We show that in a semantic categorization task with cross-domain priming, the repetition of an identity prime-target resulted in longer RTs in comparison to the repetition of an associative prime-target pair, which is suggestive of cumulative semantic interference.

In spite of showing the differential effect in RTs for association and identity words, we showed that this difference cannot be attributed to the speed of evidence accumulation. Traditionally, semantic priming effects have been explained in terms of memory aliasing, a process that helps to integrate contextual linguistic information from the prime with the visual target – see the spreading-activation theory of semantic processing (Collins and Loftus, 1975) and the compound-cue account (Ratcliff et al., 1988). According to the spreading-activation theory, semantic memory can be seen as a network of interconnected nodes. If two nodes share semantic features, they are connected, and the semantic distance determines the strength of this connection. This theory predicts that identity primes (i.e., the words “man” and “woman”) would lead to a greater accessibility of the target in memory in comparison to the associative primes. Here, however, we found that the drift rate does not change as a function of semantic distance, which indicates that the drift rate does not necessarily reflect lexico-semantic memory effects but rather reflects metacognitive processes (see discussion of drift rate and meta-cognitive processes in “*Words facilitate information processing of the target picture”*). Previous experimental evidence suggests that briefly presented primes influence behavior via meta-cognitive fluency heuristics (Whittlesea and Williams, 1998, 2000). Fluency, or the meta-cognitive experience of the ease with which we process information, affects a wide variety of decisions (categorization: Oppenheimer and Frank, 2008; familiarity: Monin, 2003; and lexical decisions: Potter et al., 2018). In a general sense, decisions can be made not only on the basis of the content but also on the basis of the feeling of how easy it is to make a decision – in this sense, fluency operates as a heuristic that facilitates decision making (Schwarz, 2004).

In summary, we show that both identity and associative words increase the processing speed of the visual target. The fact that processing speed does not reflect differences in RT attributable to a semantic cumulative effect suggests that drift rate does not reflect lexico-semantic processes in this case.

### Effects of Words on Processing Speed of Gendered Faces

The facilitation of informational target processing could reflect target-specific effects related to gender given the social nature of the stimuli. To investigate this aspect, we performed an additional analysis considering the effect of words separately for male and female faces. While a facilitation effect (neutral > female) for female faces was evident from both RTs and percentage of correct responses (in both identity and associative words), we found neither a facilitation nor an interference effect for male faces (RTs for identity words showed a trend toward facilitation, but this did not reach significance). In terms of cognitive modeling, we found increased drift rates indicating facilitation for both male (male > neutral) and female (female > neutral) targets. As for the non-decisional parameter T_*er*_, we found facilitation for female targets. However, we found neither facilitation nor interference for male primes – only a general difference between male and female primes. The facilitation effect that we found in congruent vs. neutral prime-target pairs was not modulated by the gender of the target face. This suggested that both male- and female-related words introduced the same amount of “fluency” toward deciding on the target category of man or woman.

In social psychology, words or bigger chunks of verbal material are typically used to study social group effects in decision making (Bodenhausen, 1988; Kunda and Sherman-Williams, 1993). For example, when reading a description of a court case that described a defendant either as Latino or White, the Latino was considered more guilty in comparison to the White by the participants (Bodenhausen, 1988). The words with which we communicate refer to various “schemata” that people use in their perception (Bartlett, 1932). By “schemata” we mean a set of features/beliefs that subjectively reflect the social category of gender and subsequently influence our decisions. Put differently, language can introduce a top-down bias that can influence both visual perception (Simanova et al., 2016) and social decision making (Kunda and Sherman-Williams, 1993).

Studies investigating the effects of social category on perception propose two different mechanisms that can explain this bias: perception and executive accounts. In a weapon identification task (WIT), participants have been found to more often judge an object as a gun when the object is preceded by the face of a black person as opposed to the face of a white person. In the study by Payne et al. (2005), participants who had a chance to correct their response did almost perfectly on the task. It was proposed that the bias in performance was therefore due to the participants’ incorrect initial response. If they actually misperceived an object as a gun, then regardless of time limits, their answer would have stayed constant. This account was supported by a study by Amodio et al. (2004), who used the WIT to study stereotypic responses by examining the event-related negativity potentials recorded from the brain (ERN), which are known to reflect response conflict. They found an increased ERN response on the trials where black faces were followed by an object perceived as a gun, which suggested that participants were partially aware of the fact that they had made a mistake. Overall, the fact that people misperceive the object as a gun is explained by the failure of the executive control mechanism. On the other hand, Correll et al. (2015) showed that stereotypes can affect the speed of visual identification of the object. They used a first-person shooter task where participants saw black or white men holding either innocuous objects (e.g., a wallet) or guns and showed that the information about a gun was accumulated faster if a black man held it. To sum up, the “misperception” might have occurred for two different reasons: either because of the fluency of visual processing or the mechanisms of control.

In the field of language, a similar issue has been debated. Previous studies investigated whether semantic effects in response to priming paradigms (e.g., the words “A” or “B” are used as primes, and the participants have to make an “A” or “B” decision on the target) can be explained by response facilitation at the motor stage and/or target processing facilitation (Voss et al., 2013). In other words, it has been proposed that a prime can pre-activate a certain motor response option, bypassing target processing (see the interference-facilitation account of linguistic priming effects in De Houwer et al., 2002; Musch and Klauer, 2003). In this view, the prime can either facilitate motor execution if the upcoming target is coherent with the prime or interfere with the execution if there is a prime-target mismatch. In the current study, we found that both the speed of evidence accumulation and the non-decisional time were affected by the primes. This suggests that the primes do not bypass the evaluation of the target but rather exert their influence on the target, thus ruling out the interference-facilitation account. In conclusion, in this study, we found that both associations and identity words in response priming led to a facilitation of face gender categorization. This effect was mapped to both response and target processing: when related to the target, both prime types resulted in increased processing speed and faster motor response preparation. This result highlights the multidimensionality of the cognitive processes affected by language.

## Data Availability Statement

All datasets generated for this study are included in the article/Supplementary Material.

## Ethics Statement

This study was carried out in accordance with the recommendations of CMO Arnhem-Nijmegen, Radboud University Medical Center ethical committee with written informed consent from all subjects. All subjects gave written informed consent in accordance with the Declaration of Helsinki. The protocol was approved by the CMO Arnhem-Nijmegen, Radboud University Medical Center.

## Author Contributions

LT conceptualized and designed the experiment and acquired the data. Both authors analyzed the data, wrote the article, approved the final version of the article, and agreed to be accountable for all aspects of this work.

## Conflict of Interest

The authors declare that the research was conducted in the absence of any commercial or financial relationships that could be construed as a potential conflict of interest.

## References

[B1] Amado C. Kovács P. Mayer R. Ambrus G. G. Trapp S. Kovács G. (2018). Neuroimaging results suggest the role of prediction in cross-domain priming. *Sci. Rep.* 8:10356. 10.1038/s41598-018-28696-0 29985455 PMC6037787

[B2] Amodio D. M. Harmon-Jones E. Devine P. G. Curtin J. J. Hartley S. L. Covert A. E. (2004). Neural signals for the detection of unintentional race bias. *Psychol. Sci.* 15, 88–93. 10.1111/j.0963-7214.2004.01502003.x 14738514

[B3] Anderson D. E. Serences J. T. Vogel E. K. Awh E. (2014). Induced alpha rhythms track the content and quality of visual working memory representations with high temporal precision. *J. Neurosci.* 34 7587–7599. 10.1523/JNEUROSCI.0293-14.2014 24872563 PMC4035520

[B4] Bartlett F. C. (1932). *Remembering.* London: Cambridge University Press.

[B5] Blanz V. Vetter T. (1999). “A morphable model for the synthesis of 3D faces,” in *Proceedings of the 26th Annual Conference on Computer Graphics and Interactive Techniques - SIGGRAPH ’99*, (New York, NY: ACM Press), 187–194. 10.1145/311535.311556

[B6] Bodenhausen G. V. (1988). Stereotypic biases in social decision making and memory: testing process models of stereotype use. *J. Pers. Soc. Psychol.* 55 726–737. 10.1037/0022-3514.55.5.726 3210142

[B7] Bogacz R. Wagenmakers E. J. Forstmann B. U. Nieuwenhuis S. (2010). The neural basis of the speed-accuracy tradeoff. *Trends Neurosci.* 33 10–16. 10.1016/j.tins.2009.09.002 19819033

[B8] Boutonnet B. Lupyan G. (2015). Words jump-start vision: a label advantage in object recognition. *J. Neurosci.* 35 9329–9335. 10.1523/JNEUROSCI.5111-14.2015 26109657 PMC6605198

[B9] Burnham K. P. Anderson D. R. Burnham K. P. (2002). *Model Selection and Multimodel Inference: A Practical Information-Theoretic Approach*. Berlin: Springer.

[B10] Campanella F. Crescentini C. Mussoni A. Skrap M. (2013). Refractory semantic access dysphasia resulting from resection of a left frontal glioma. *Neurocase* 19 27–35. 10.1080/13554794.2011.654212 22519645

[B11] Carroll N. C. Young A. W. (2005). Priming of emotion recognition. *Q. J. Exp. Psychol. Sec. A* 58 1173–1197. 10.1080/02724980443000539 16194954

[B12] Cohen J. D. Dunbar K. McClelland J. L. (1990). On the control of automatic processes: a parallel distributed processing account of the Stroop effect. *Psychol. Rev.* 97 332–361. 10.1037/0033-295x.97.3.332 2200075

[B13] Collins A. M. Loftus E. F. (1975). A spreading-activation theory of semantic processing. *Psychol. Rev.* 82 407–428. 10.1037/0033-295X.82.6.407

[B14] Correll J. Wittenbrink B. Crawford M. T. Sadler M. S. (2015). Stereotypic vision: how stereotypes disambiguate visual stimuli. *J. Pers. Soc. Psychol.* 108 219–233. 10.1037/pspa0000015 25603373

[B15] De Deyne S. Navarro D. J. Storms G. (2013). Better explanations of lexical and semantic cognition using networks derived from continued rather than single-word associations. *Behav. Res. Methods* 45 480–498. 10.3758/s13428-012-0260-7 23055165

[B16] De Houwer J. Hermans D. Rothermund K. Wentura D. (2002). Affective priming of semantic categorisation responses. *Cogn. Emot.* 16 643–666. 10.1080/02699930143000419

[B17] Duncan-Johnson C. Kopell B. (1981). The Stroop effect: brain potentials localize the source of interference. *Science* 214 938–940. 10.1126/science.7302571 7302571

[B18] Duncan-Johnson C. C. (1981). Young Psychophysiologist Award address, 1980. P300 latency: a new metric of information processing. *Psychophysiology* 18 207–215. 10.1111/psyp.1981.18.issue-3 7291436

[B19] Dunovan K. E. Tremel J. J. Wheeler M. E. (2014). Prior probability and feature predictability interactively bias perceptual decisions. *Neuropsychologia* 61 210–221. 10.1016/j.neuropsychologia.2014.06.024 24978303 PMC4126168

[B20] Faria C. de A. Alves H. V. D. Charchat-Fichman H. (2015). The most frequently used tests for assessing executive functions in aging. *Dement. Neuropsychol.* 9 149–155. 10.1590/1980-57642015DN92000009 29213956 PMC5619353

[B21] Francken J. C. Kok P. Hagoort P. de Lange F. P. (2015). The behavioral and neural effects of language on motion perception. *J. Cogn. Neurosci.* 27 175–184. 10.1162/jocn_a_00682 25000524

[B22] Gelman A. Rubin D. B. (1992). Inference from iterative simulation using multiple sequences. *Stat. Sci.* 7, 457–472. 10.1214/ss/1177011136

[B23] Gilbert A. L. Regier T. Kay P. Ivry R. B. (2006). Whorf hypothesis is supported in the right visual field but not the left. *Proc. Natl. Acad. Sci. U.S.A.* 103 489–494. 10.1073/pnas.0509868103 16387848 PMC1326182

[B24] Gilbert A. L. Regier T. Kay P. Ivry R. B. (2008). Support for lateralization of the Whorf effect beyond the realm of color discrimination. *Brain Lang.* 105 91–98. 10.1016/j.bandl.2007.06.001 17628656

[B25] Glaser W. R. Düngelhoff F. J. (1984). The time course of picture-word interference. *J. Exp. Psychol.* 10 640–654. 10.1037/0096-1523.10.5.640 6238124

[B26] Gomez P. Perea M. Ratcliff R. (2013). A diffusion model account of masked versus unmasked priming: are they qualitatively different? *J. Exp. Psychol.* 39 1731–1740. 10.1037/a0032333 23647337 PMC5688948

[B27] JASP Team (2018). *JASP (Version 0.8.6)[Computer software].* Amsterdam: JASP Team.

[B28] Jefferies E. Patterson K. Ralph M. A. L. (2008). Deficits of knowledge versus executive control in semantic cognition: insights from cued naming. *Neuropsychologia* 46 649–658. 10.1016/j.neuropsychologia.2007.09.007 17961610 PMC2350189

[B29] Keuleers E. Brysbaert M. New B. (2010). SUBTLEX-NL: a new measure for Dutch word frequency based on film subtitles. *Behav. Res. Methods* 42 643–650. 10.3758/BRM.42.3.643 20805586

[B30] Kinoshita S. de Wit B. Aji M. Norris D. (2017). Evidence accumulation in the integrated and primed Stroop tasks. *Mem. Cogn.* 45 824–836. 10.3758/s13421-017-0701-8 28364405 PMC5498641

[B31] Kuipers J.-R. La Heij W. (2008). Semantic facilitation in category and action naming: testing the message-congruency account. *J. Mem. Lang.* 58 123–139. 10.1016/j.jml.2007.05.005

[B32] Kunda Z. Sherman-Williams B. (1993). Stereotypes and the Construal of Individuating Information. *Pers. Soc. Psychol. Bull.* 19 90–99. 10.1177/0146167293191010

[B33] Kutas M. Federmeier K. D. (2011). Thirty years and counting: finding meaning in the N400 component of the event-related brain potential (ERP). *Annu. Rev. Psychol.* 62 621–647. 10.1146/annurev.psych.093008.131123 20809790 PMC4052444

[B34] Kutas M. McCarthy G. Donchin E. (1977). Augmenting mental chronometry: the P300 as a measure of stimulus evaluation time. *Science* 197 792–795. 10.1126/science.887923 887923

[B35] Landau A. N. Aziz-Zadeh L. Ivry R. B. (2010). The influence of language on perception: listening to sentences about faces affects the perception of faces. *J. Neurosci.* 30 15254–15261. 10.1523/JNEUROSCI.2046-10.2010 21068330 PMC6633823

[B36] Lupker S. J. (1979). The semantic nature of response competition in the picture-word interference task. *Mem. Cogn.* 7 485–495. 10.3758/bf03198265

[B37] Madden D. J. Siciliano R. E. Tallman C. W. Monge Z. A. Voss A. Cohen J. R. (2019). Response-level processing during visual feature search: effects of frontoparietal activation and adult age. *Attent. Percept. Psychophys.* 82 330–349. 10.3758/s13414-019-01823-3 31376024 PMC6995405

[B38] Mahon B. Z. Costa A. Peterson R. Vargas K. A. Caramazza A. (2007). Lexical selection is not by competition: a reinterpretation of semantic interference and facilitation effects in the picture-word interference paradigm. *J. Exp. Psychol.* 33 503–535. 10.1037/0278-7393.33.3.503 17470003

[B39] Mandera P. Keuleers E. Brysbaert M. (2017). Explaining human performance in psycholinguistic tasks with models of semantic similarity based on prediction and counting: a review and empirical validation. *J. Mem. Lang.* 92 57–78. 10.1016/j.jml.2016.04.001

[B40] Meteyard L. Bahrami B. Vigliocco G. (2007). Motion detection and motion verbs. *Psychol. Sci.* 18 1007–1013. 10.1111/j.1467-9280.2007.02016.x 17958716

[B41] Mikolov T. Chen K. Corrado G. Dean J. (2013). “Efficient estimation of word representations in vector space,” in *Proceedings of the 1st International Conference on Learning Representations, ICLR 2013 - Workshop Track Proceedings. International Conference on Learning Representations*, (Scottsdale: ICLR).

[B42] Mirman D. Landrigan J.-F. Britt A. E. (2017). Taxonomic and thematic semantic systems. *Psychol. Bull.* 143 499–520. 10.1037/bul0000092 28333494 PMC5393928

[B43] Monin B. (2003). The warm glow heuristic: when liking leads to familiarity. *J. Pers. Soc. Psychol.* 85 1035–1048. 10.1037/0022-3514.85.6.1035 14674812

[B44] Mulder M. J. van Maanen L. Forstmann B. U. (2014). Perceptual decision neurosciences – A model-based review. *Neuroscience* 277 872–884. 10.1016/j.neuroscience.2014.07.031 25080159

[B45] Mulder M. J. Wagenmakers E. J. Ratcliff R. Boekel W. Forstmann B. U. (2012). Bias in the brain: a diffusion model analysis of prior probability and potential payoff. *J. Neurosci.* 32 2335–2343. 10.1523/JNEUROSCI.4156-11.2012 22396408 PMC6621823

[B46] Musch J. Klauer K. C. (eds). (2003). *The Psychology of Evaluation: Affective Processes in Cognition and Emotion – Google Boeken.* Hove: Psychology Press Available online at: https://books.google.nl/books?hl=nl&lr =&id=t1h6AgAAQBAJ&oi=fnd&pg=PA9&dq=klauer+2003+affective+priming &ots=bBIA87KF4_&sig=rC41jScrG1-KpROOfSwyNRpY69c# v = onepage&q = klauer2003 affective priming&f=false

[B47] Navarrete E. Mahon B. Z. Caramazza A. (2010). The cumulative semantic cost does not reflect lexical selection by competition. *Acta Psychol.* 134 279–289. 10.1016/j.actpsy.2010.02.009 20347062 PMC2908277

[B48] Nunez M. D. Gosai A. Vandekerckhove J. Srinivasan R. (2019). The latency of a visual evoked potential tracks the onset of decision making. *Neuroimage* 197 93–108. 10.1016/j.neuroimage.2019.04.052 31028925

[B49] Oppenheim G. M. Dell G. S. Schwartz M. F. (2007). Cumulative semantic interference as learning. *Brain Lang.* 103 175–176. 10.1016/j.bandl.2007.07.102

[B50] Oppenheimer D. M. Frank M. C. (2008). A rose in any other font would not smell as sweet: effects of perceptual fluency on categorization. *Cognition* 106 1178–1194. 10.1016/j.cognition.2007.05.010 17618616

[B51] Payne B. K. Shimizu Y. Jacoby L. L. (2005). Mental control and visual illusions: toward explaining race-biased weapon misidentifications. *J. Exp. Soc. Psychol.* 41 36–47. 10.1016/j.jesp.2004.05.001

[B52] Piai V. Knight R. T. (2018). Lexical selection with competing distractors: evidence from left temporal lobe lesions. *Psychonom. Bull. Rev.* 25 710–717. 10.3758/s13423-017-1301-0 28484950 PMC5902514

[B53] Piai V. Roelofs A. Acheson D. J. Takashima A. (2013). Attention for speaking: domain-general control from the anterior cingulate cortex in spoken word production. *Front. Hum. Neurosci.* 7:832. 10.3389/fnhum.2013.00832 24368899 PMC3856851

[B54] Potter K. W. Donkin C. Huber D. E. (2018). The elimination of positive priming with increasing prime duration reflects a transition from perceptual fluency to disfluency rather than bias against primed words. *Cogn. Psychol.* 101 1–28. 10.1016/j.cogpsych.2017.11.004 29241033

[B55] Ratcliff R. McKoon G. (2008). The diffusion decision model: theory and data for two-choice decision tasks. *Neural Comput.* 20 873–922. 10.1162/neco.2008.12-06-420 18085991 PMC2474742

[B56] Ratcliff R. McKoon G. Hintzman D. Mandler G. Masson M. Jo Nissen M. (1988). A retrieval theory of priming in memory. *Psychol. Rev.* 95 385–408. 10.1037/0033-295x.95.3.385 3406246

[B57] Samaha J. Boutonnet B. Postle B. R. Lupyan G. (2018). Effects of meaningfulness on perception: alpha-band oscillations carry perceptual expectations and influence early visual responses. *Sci. Rep.* 8:6606. 10.1038/s41598-018-25093-5 29700428 PMC5920106

[B58] Schwarz G. (1978). Estimating the dimension of a model. *Ann. Stat.* 6, 461–464. 10.1214/aos/1176344136

[B59] Schwarz N. (2004). Metacognitive experiences in consumer judgment and decision making. *J. Consumer Psychol.* 14 332–348. 10.1207/s15327663jcp1404_2

[B60] Simanova I. Francken J. C. de Lange F. P. Bekkering H. (2016). Linguistic priors shape categorical perception. *Lang. Cogn. Neurosci.* 31 159–165. 10.1080/23273798.2015.1072638

[B61] Smith P. L. Ratcliff R. (2004). Psychology and neurobiology of simple decisions. *Trends Neurosci.* 27 161–168. 10.1016/j.tins.2004.01.006 15036882

[B62] Tagliabue C. F. Veniero D. Benwell C. S. Y. Cecere R. Savazzi S. Thut G. (2019). The EEG signature of sensory evidence accumulation during decision formation closely tracks subjective perceptual experience. *Sci. Rep.* 9:4949. 10.1038/s41598-019-41024-4 30894558 PMC6426990

[B63] Twomey D. M. Murphy P. R. Kelly S. P. O’Connell R. G. (2015). The classic P300 encodes a build-to-threshold decision variable. *Eur. J. Neurosci.* 42 1636–1643. 10.1111/ejn.12936 25925534

[B64] Usher M. McClelland J. L. (2001). The time course of perceptual choice: the leaky, competing accumulator model. *Psychol. Rev.* 108 550–592. 10.1037/0033-295x.108.3.550 11488378

[B65] Voss A. Rothermund K. Gast A. Wentura D. (2013). Cognitive processes in associative and categorical priming: a diffusion model analysis. *J. Exp. Psychol.* 142 536–559. 10.1037/a0029459 22866687

[B66] Voss A. Rothermund K. Voss J. (2004). Interpreting the parameters of the diffusion model: an empirical validation. *Mem. Cogn.* 32 1206–1220. 10.3758/bf03196893 15813501

[B67] Wagenmakers E.-J. Marsman M. Jamil T. Ly A. Verhagen J. Love J. (2018). Bayesian inference for psychology. Part I: Theoretical advantages and practical ramifications. *Psychon. Bull. Rev.* 25, 35–57. 10.3758/s13423-017-1343-3 28779455 PMC5862936

[B68] Wagenmakers E. J. Van Der Maas H. L. J. Grasman R. P. P. P. (2007). An EZ-diffusion model for response time and accuracy. *Psychonomic Bulletin and Review* 14 3–22. 10.3758/BF03194023 17546727

[B69] Wentura D. Degner J. (2010). “A practical guide to sequential priming and related tasks,” in *Handbook of Implicit Social Cognition: Measurement, Theory and Applications*, eds Gawronski B. Payne B. K. (New York, NY: Guilford Press), 95–115.

[B70] Whittlesea B. W. A. Williams L. D. (1998). Why do strangers feel familiar, but friends don’t? A discrepancy-attribution account of feelings of familiarity. *Acta Psychol.* 98 141–165. 10.1016/s0001-6918(97)00040-1 9621828

[B71] Whittlesea B. W. A. Williams L. D. (2000). The source of feelings of familiarity: the discrepancy-attribution hypothesis. *J. Exp. Psychol.* 26 547–565. 10.1037/0278-7393.26.3.547 10855417

[B72] Wiecki T. V. Sofer I. Frank M. J. (2013). HDDM: Hierarchical bayesian estimation of the drift-diffusion model in python. *Front. Neuroinform.* 7:14. 10.3389/fninf.2013.00014 23935581 PMC3731670

[B73] Willenbockel V. Sadr J. Fiset D. Horne G. O. Gosselin F. Tanaka J. W. (2010). Controlling low-level image properties: the SHINE toolbox. *Behav. Res. Methods* 42 671–684. 10.3758/BRM.42.3.671 20805589

[B74] Winawer J. Witthoft N. Frank M. C. Wu L. Wade A. R. Boroditsky L. (2007). Russian blues reveal effects of language on color discrimination. *Proc. Natl. Acad. Sci. U.S.A.* 104 7780–7785. 10.1073/pnas.0701644104 17470790 PMC1876524

