# Supplementary material for: Associative and Identity Words Promote the Speed of Visual Categorization: A Hierarchical Drift Diffusion Account
Source: Front Psychol. 2020 Jul 23;11:955. doi: 10.3389/fpsyg.2020.00955 (PMC7390986; doi:10.3389/fpsyg.2020.00955)
Supplement: Supplementary file 1 [file Data_Sheet_1.docx]

**SUPPLEMENTARY MATERIAL**

| **Words related to male concept** | **Words related to female concept** | **Neutral words** |
| --- | --- | --- |
| vader (father) baard (beard)  jongen (young man) snor (moustache) kostuum (costume) hoed (hat)  broer (father) zwaard (sword) kerel (guy) farao (pharaoh) stropdas (tie)  bruidegom (groom) geweer (weapon) vlinderdas (bow tie) sik (beard)  zoon (son) pijp (pipe)  smoking (tuxedo) kilt (kilt) scheermes (razor) | zus (sister)  dochter (daughter)  tante (aunt)  mama (mother) echtgenote (wife) rok (skirt)  taille (blouse)  nicht (niece)  parfum (perfume) dame (lady)  handtas (handbag)  hoofddoek (headscarf) mascara (mascara)  pruik (wig)  rouge (rouge) jurk (dress) meisje (girl)  haarstukje (hairpiece) panty (tricot) mantelpak (suit) | dag (dag)  adrenaline (adrenaline)  appelsap (apple juice)  telefoon (phone)  friten (fries)  loterij (lottery) ticket (ticket) tunnel (tunnel) verlof (vacation) gazon (lawn) koord(string) rivier (river)  vuur (fire)  roem (fame)  maag (stomach) bos (forest)  pen (pen)  kiosk (kiosk)  bioscoop (cinema) rekening (bill) |

**Table S1.1.** List of associative words related to male and female concepts and neutral words.

| **Prime count (associations before division)** | **prime** | **target** | **Cosine distance** | **Prime type (division of associative)** |
| --- | --- | --- | --- | --- |
| 1 | vader | man | 0.471586 | Label |
| 2 | baard | man | 0.67291 | Association |
| 3 | jongen | man | 0.347639 | Label |
| 4 | snor | man | 0.669841 | Association |
| 5 | kostuum | man | 0.763772 | Association |
| 6 | hoed | man | 0.696146 | Association |
| 7 | broer | man | 0.465685 | Label |
| 8 | zwaard | man | 0.661439 | Association |
| 9 | kerel | man | 0.274264 | Label |
| 10 | farao | man | 0.802513 | Label |
| 11 | stropdas | man | 0.709387 | association |
| 12 | bruidegom | man | 0.7429 | Label |
| 13 | geweer | man | 0.610479 | Association |
| 14 | vlinderdas | man | 0.762285 | Association |
| 15 | sik | man | 0.754113 | Association |
| 16 | zoon | man | 0.45961 | Label |
| 17 | pijp | man | 0.734082 | Association |
| 18 | smoking | man | 0.720216 | Association |
| 19 | kilt | man | 0.677306 | association |
| 20 | scheermes | man | 0.659975 | association |
| 1 | zus | vrouw | 0.472862 | Label |
| 2 | dochter | vrouw | 0.311131 | Label |
| 3 | tante | vrouw | 0.537335 | Label |
| 4 | mama | vrouw | 0.640638 | Label |
| 5 | echtgenote | vrouw | 0.363388 | Label |
| 6 | rok | vrouw | 0.750863 | Association |
| 7 | taille | vrouw | 0.800168 | Association |
| 8 | nicht | vrouw | 0.647979 | Label |
| 9 | parfum | vrouw | 0.690007 | Association |
| 10 | dame | vrouw | 0.452672 | Label |
| 11 | handtas | vrouw | 0.638115 | Association |
| 12 | hoofddoek | vrouw | 0.821153 | Association |
| 13 | mascara | vrouw | 0.766208 | Association |
| 14 | pruik | vrouw | 0.682367 | Association |
| 15 | rouge | vrouw | 0.823189 | Association |
| 16 | jurk | vrouw | 0.53669 | Association |
| 17 | meisje | vrouw | 0.43151 | Label |
| 18 | haarstukje | vrouw | 0.565204 | Association |
| 19 | panty | vrouw | 0.702756 | Association |
| 20 | mantelpak | vrouw | 0.656435 | Association |
| 1 | vrouw | vrouw | -1.19209E-007 | Identity |
| 2 | vrouw | vrouw | -1.19209E-007 | Identity |
| 3 | vrouw | vrouw | -1.19209E-007 | Identity |
| 4 | vrouw | vrouw | -1.19209E-007 | Identity |
| 5 | vrouw | vrouw | -1.19209E-007 | Identity |
| 6 | vrouw | vrouw | -1.19209E-007 | Identity |
| 7 | vrouw | vrouw | -1.19209E-007 | Identity |
| 8 | vrouw | vrouw | -1.19209E-007 | Identity |
| 9 | vrouw | vrouw | -1.19209E-007 | Identity |
| 10 | vrouw | vrouw | -1.19209E-007 | Identity |
| 11 | vrouw | vrouw | -1.19209E-007 | Identity |
| 12 | vrouw | vrouw | -1.19209E-007 | Identity |
| 13 | vrouw | vrouw | -1.19209E-007 | Identity |
| 14 | vrouw | vrouw | -1.19209E-007 | Identity |
| 15 | vrouw | vrouw | -1.19209E-007 | Identity |
| 16 | vrouw | vrouw | -1.19209E-007 | Identity |
| 17 | vrouw | vrouw | -1.19209E-007 | Identity |
| 18 | vrouw | vrouw | -1.19209E-007 | Identity |
| 19 | vrouw | vrouw | -1.19209E-007 | Identity |
| 20 | vrouw | vrouw | -1.19209E-007 | Identity |
| 1 | man | man | 0 | Identity |
| 2 | man | man | 0 | Identity |
| 3 | man | man | 0 | Identity |
| 4 | man | man | 0 | Identity |
| 5 | man | man | 0 | Identity |
| 6 | man | man | 0 | Identity |
| 7 | man | man | 0 | Identity |
| 8 | man | man | 0 | Identity |
| 9 | man | man | 0 | Identity |
| 10 | man | man | 0 | Identity |
| 11 | man | man | 0 | Identity |
| 12 | man | man | 0 | Identity |
| 13 | man | man | 0 | Identity |
| 14 | man | man | 0 | Identity |
| 15 | man | man | 0 | Identity |
| 16 | man | man | 0 | Identity |
| 17 | man | man | 0 | Identity |
| 18 | man | man | 0 | Identity |
| 19 | man | man | 0 | Identity |
| 20 | man | man | 0 | Identity |

**Table S1.2.** Similarity measures in terms of cosine distance derived from word2vec model and proposed division of associative words into purely associative and labels (Prime type column).

| **Model subset** | **v** | **A** | **T_er_** | **DIC** |
| --- | --- | --- | --- | --- |
| **0** | **C/T** | **C/T** | **C/T** | **-16537.9** |
| **1** | **C** | **C/T** | **C/T** | **-16549.3** |
| **2** | **C/T** | **C** | **C/T** | **-16485.2** |
| **3** | **C/T** | **C/T** | **C** | **-16080.9** |
| **4** | **C** | **C** | **C** | **-16547.9** |
| **5** | **T** | **C/T** | **C/T** | **-16515.5** |
| **6** | **C/T** | **T** | **C/T** | **-16601.7** |
| **7** | **C/T** | **C/T** | **T** | **-16110.6** |
| **8** | **T** | **T** | **T** | **-16147.8** |
| **9** | **C** | **C** | **C/T** | **-16496.3** |
| **10** | **C** | **C/T** | **C** | **-16114.7** |
| **11** | **C/T** | **C** | **C** | **-15890.1** |
| **12** | **T** | **T** | **C/T** | **-16588.0** |
| **13** | **T** | **C/T** | **T** | **-16074.3** |
| **14** | **C/T** | **T** | **T** | **-16213.7** |

**Table S2. List of models for the congruency analysis.** Deviance information criterion scores (DIC) and fixed parameters for all models with C = congruency, T = prime type.


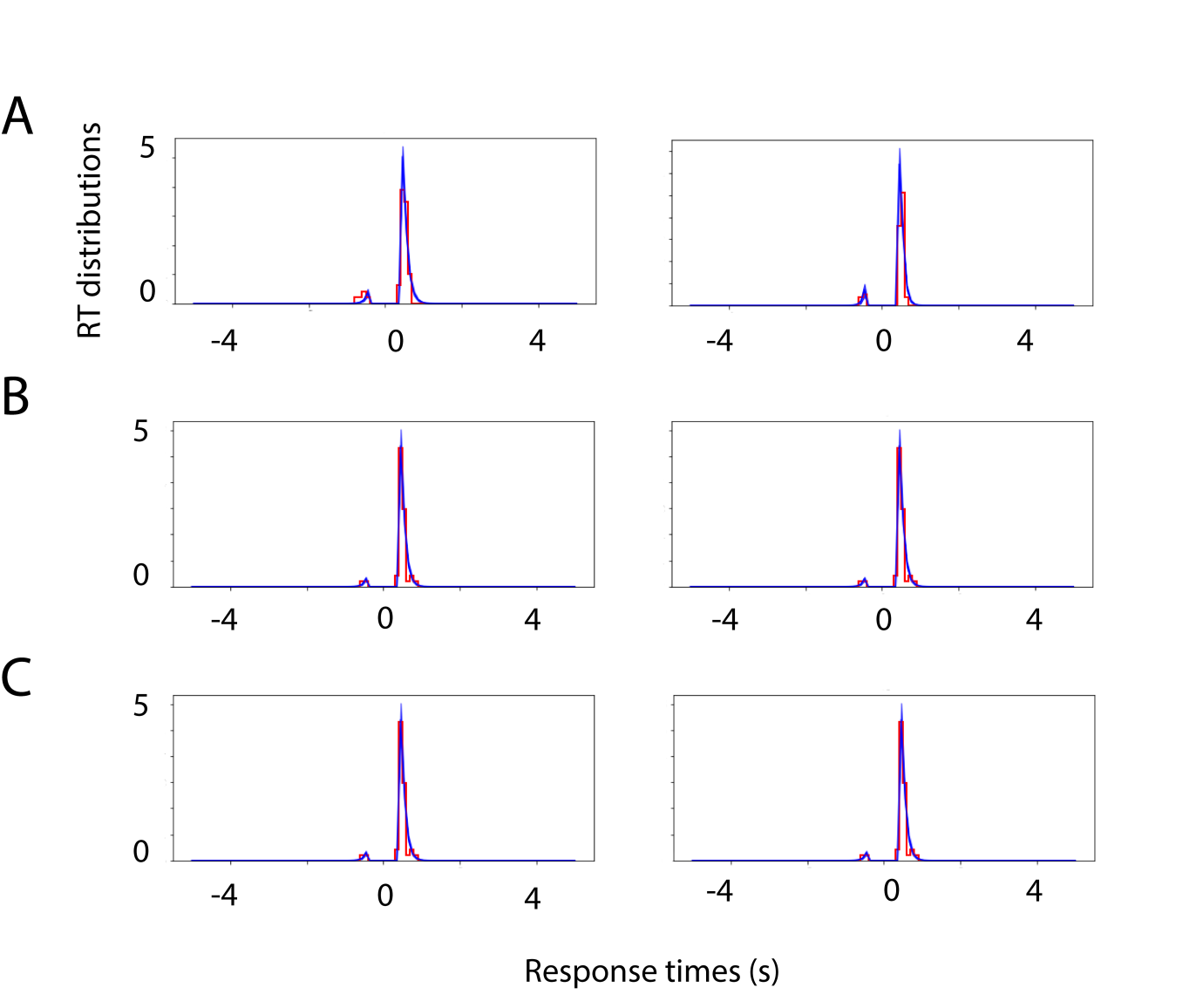


**Figure S1: Congruency analysis:** Observed RT distributions (red lines) and predicted posteriors (blue line) for the drift rate for subject number 10 (A left – congruent associative, A right – congruent identity; B left – incongruent associative, B right – incongruent identity; C left – neutral associative, C right – neutral identity).

| **Model subset** | **v** | **A** | **T_er_** | **DIC** |
| --- | --- | --- | --- | --- |
| **1** | **PTS** | **PTS** | **PTS** | **-17571.3** |
| **2** | **PT** | **PTS** | **PTS** | **-17577.5** |
| **3** | **PTS** | **PT** | **PTS** | **-17569.4** |
| **4** | **PTS** | **PTS** | **PT** | **-17571.8** |
| **5** | **PT** | **PT** | **PT** | **-17582.7** |
| **6** | **PS** | **PTS** | **PTS** | **-17620.5** |
| **7** | **PTS** | **PS** | **PTS** | **-17553.7** |
| **8** | **PTS** | **PTS** | **PS** | **-16988** |
| **9** | **PS** | **PS** | **PS** | **-16786.7** |
| **10** | **TS** | **PTS** | **PTS** | **-17607.6** |
| **11** | **PTS** | **TS** | **PTS** | **-17617.7** |
| **12** | **PTS** | **PTS** | **TS** | **-16910.4** |
| **13** | **TS** | **TS** | **TS** | **-17205** |
| **14** | **P** | **PT** | **PT** | **-17111.1** |
| **15** | **PT** | **P** | **PT** | **-17489** |
| **16** | **PT** | **PT** | **P** | **-16467.8** |
| **17** | **P** | **PS** | **PS** | **-16252.2** |
| **18** | **PS** | **P** | **PS** | **-16671.3** |
| **19** | **PS** | **PS** | **P** | **-16303.9** |
| **20** | **P** | **P** | **P** | **-15662.3** |
| **21** | **T** | **T** | **T** | **-16146.7** |
| **22** | **S** | **S** | **S** | **-16406.6** |
| **23** | **PS** | **PT** | **PTS** | **-17618.3** |
| **24** | **PS** | **PTS** | **PT** | **-17627.3** |
| **25** | **PS** | **PT** | **PT** | **-17627** |
| **26** | **PS** | **PS** | **PTS** | **-17586.8** |
| **27** | **PS** | **PTS** | **PS** | **-16989.7** |
| **28** | **PS** | **P** | **PTS** | **-17488.8** |
| **29** | **PS** | **PTS** | **P** | **-16508** |
| **30** | **PS** | **TS** | **PTS** | **-17677.7** |
| **31** | **PS** | **PTS** | **TS** | **-16951.3** |
| **32** | **PS** | **TS** | **TS** | **-17185.9** |

**Table S3.** **List of models for the analysis of priming effects on face gender.** Deviance information criterion scores (DIC) and fixed parameters for all models with: P = prime gender, T = prime type, S = target stimulus.

| **Model subset** | **v** | **A** | **T_er_** | **z** | **DIC** |
| --- | --- | --- | --- | --- | --- |
| **30** | **PS** | **TS** | **PTS** | **sex** | **-17677.7** |
| **30A** | **PS** | **TS** | **PTS** | **sex** | **-17.674** |
| **30B** | **PS** | **TS** | **PTS** | **-** | **-17.382** |

**Table S4.** **List of models for the analysis of priming effects on face gender with stratified bias**. Deviance information criterion scores (DIC) and fixed parameters for all models with: P = prime gender, T = prime type, S = target stimulus, sex = sex of subjects.
